# Supplementary figures and images for: The SmartSleep Experiment: Evaluation of changes in night-time smartphone behavior following a mass media citizen science campaign
Source: PLoS One. 2021 Jul 21;16(7):e0253783. doi: 10.1371/journal.pone.0253783 (PMC8294485; doi:10.1371/journal.pone.0253783)

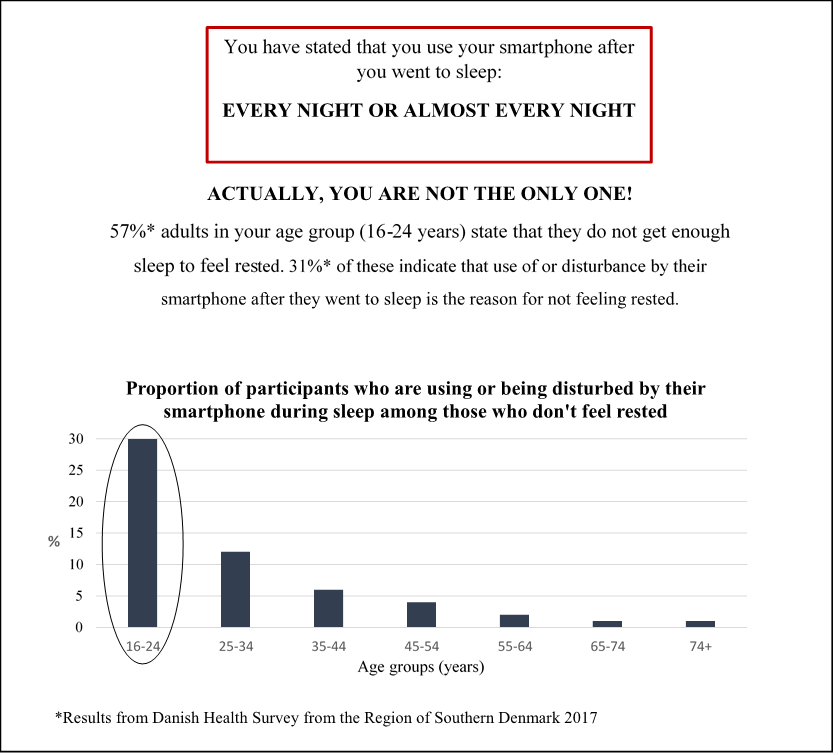

Supplement: S1 Fig — (TIFF) [file pone.0253783.s001.tiff]
