# Supplementary material for: The SmartSleep Experiment: Evaluation of changes in night-time smartphone behavior following a mass media citizen science campaign
Source: PLoS One. 2021 Jul 21;16(7):e0253783. doi: 10.1371/journal.pone.0253783 (PMC8294485; doi:10.1371/journal.pone.0253783)
Supplement: S1 Table — (PDF) [file pone.0253783.s003.pdf]

S1 Table: Characteristics of individuals who participated in the follow-up study and individuals who did not participate in the follow-up study

	**Individuals who did not agree to participate in follow-up study** N=12,887	**Individuals who agreed at baseline but did not participate in follow-up study** N=3,337	**Individuals who participated in follow-up study** N=8,911
**Age,** mean (SD)	41.6 (15)	42.3 (15.2)	45 (15)
**Female,** %	62	64	61
**Educational level**[a], %			
Low	9	7	4
Medium	25	29	23
High	63	53	71
Other	2	2	2
**Occupational status,** %			
Employed or self-employed	64	65	66
Student	18	17	14
Unemployed	3	3	3
Outside labor market	10	10	13
Long-term sick leave	2	1	1
Other	3	4	4
**Living alone,** %	19	20	22
**Baseline night-time smartphone users**[b], %	59	64	55
**Sleep quality,** mean (SD)	2.7 (0.9)	2.9 (1)	2.8 (1)

[a]Low education: Primary school, medium education: Upper secondary school or technical vocational education, high education: Short, medium, or high cycle higher education
[b] Reporting ‘every night or almost every night’, ‘several nights a week’ or, ’several nights a month or less’
