# Supplementary material for: The SmartSleep Experiment: Evaluation of changes in night-time smartphone behavior following a mass media citizen science campaign
Source: PLoS One. 2021 Jul 21;16(7):e0253783. doi: 10.1371/journal.pone.0253783 (PMC8294485; doi:10.1371/journal.pone.0253783)
Supplement: S2 Table — (PDF) [file pone.0253783.s004.pdf]

S2 Table. Baseline night-time smartphone use and socio-demographic factors

	**Baseline night-time smartphone user**		
	Yes[b]	No[c]	p-value
	N=4,926 (55)	N=3,968 (45)	
**Educational level**[a]			
Low	237 (5)	143 (4)	
Medium	1,194 (24)	848 (21)	
High	3,399 (69)	2,884 (73)	
Other	96 (2)	93 (2)	<0.001
**Occupational status**			
Employed	3,182 (65)	2,671 (67)	
Student	939 (19)	279 (7)	
Unemployed	146 (3)	87 (2)	
Outside labor market	379 (8)	765 (19)	
Long-term sick leave	84 (2)	35 (1)	
Other	196 (4)	131 (3)	<0.001

[a] Low education: Primary school; Medium education: Upper secondary school or technical vocational education; High education: Short, medium, or high cycle higher education

[b] Reporting' every night or almost every night', 'several nights a week or more', or 'several nights a month or less'

[c] Reporting 'never'
