# Supplementary material for: The SmartSleep Experiment: Evaluation of changes in night-time smartphone behavior following a mass media citizen science campaign
Source: PLoS One. 2021 Jul 21;16(7):e0253783. doi: 10.1371/journal.pone.0253783 (PMC8294485; doi:10.1371/journal.pone.0253783)
Supplement: Attachment — Submitted filename: Response to reviewers.docx [file pone.0253783.s006.docx]

**Editor’s comment:**

Please review your reference list to ensure that it is complete and correct. If you have cited papers that have been retracted, please include the rationale for doing so in the manuscript text, or remove these references and replace them with relevant current references. Any changes to the reference list should be mentioned in the rebuttal letter that accompanies your revised manuscript. If you need to cite a retracted article, indicate the article’s retracted status in the References list and also include a citation and full reference for the retraction notice.

Response: We have made some corrections to the reference list, which should now be complete and correct.

**Reviewer #2**

I appreciate the revisions the authors made to improve the clarity of the methods and address study limitations. For the most part, the authors adequately responded to my comments. The few exceptions are as follows:

1. In my opinion, the most substantial limitation is that there was no risk-adjustment made based on baseline nightly smartphone use when reporting results of the intervention and demographic differences. For instance, 40% of participants who regularly use their smartphone at night changed or attempted to change habits relative to 11% of respondents who used their smartphone at night several nights a month or less. These data suggest baseline nightly smartphone use is a large predictor of whether or not people attempt change. The second paragraph of the results now mentions differences in the outcome as well as sociodemographic characteristics by baseline night-time smartphone use; this is very helpful. However, it remains unknown the extent to which differences in who is changing or attempting to change nightly smartphone habits are merely an artifact of baseline use. This is a problem particularly if the interpretation suggests differences in the effectiveness of the intervention by demographic differences, but these likely partly if not completely stem from baseline risks (e.g., do younger folks attempt/successfully change habits only because they are much more likely to initially have problems with nightly use?). Another example where this comes up is how the data are generally interpreted (e.g., "still 85% of the study population indicated that they had not changed night-time smartphone behavior), which suggests that lack of change is a failure of the intervention. Some might not have anywhere to change to in that they reported no use before. It is thus essential that some risk-adjustment is made and that the data are interpreted based on who has potential to change due to baseline problems.

Response: This is a valid point. In order to take the baseline night-time smartphone use into account in our interpretation of the results, we have restricted all analyses to the participants who report using their smartphone during sleep at baseline (n=4,926 participants). We have updated Figure 2-4 and corrected the results throughout the paper. The overall findings are unchanged.

2. Causal language is still used throughout including in the title ("interventional effects") despite this study being descriptive and susceptible to bias from sampling and self-reported measures. The descriptive nature of this study should be explicit.

Response: We changed the title accordingly: *The SmartSleep Experiment: Evaluation of changes in night-time smartphone behavior following a mass media citizen science intervention*.

Furthermore, we have included a sentence about the lack of causal inference in the discussion section lines 358-362:

*Even though we cannot determine the direct interventional effects from participating in the SmartSleep Experiment, the present findings give important insights into the connection between citizen science, mass media and interventional behavior impacts, which may be of value in future prevention strategies to improve sleep and smartphone habits.*

3. There is no recognition of treatment heterogeneity based on age. Whether or not the personal feedback was included strictly as a motivational tool, the feedback was highly age-dependent and specific to use of smartphone at night and therefore may have influenced age-specific findings in likelihood of changing nighttime smartphone use. This should at the very least be briefly mentioned as a limitation or future direction when interpreting age-specific findings.

Response: We agree, and we have mentioned this limitation in the discussion section lines 375-377:

*Furthermore, the direct personal feedback after completing the survey varied across age groups, and this may have influenced age-specific findings in likelihood of changing night-time smartphone use.*

4. Also, the language is generally clear. But, there are some grammatical errors throughout. The results could also be edited for clarity, particularly the first two paragraphs.

Response: Thank you for highlighting this. We have edited the result section and corrected the grammatical errors throughout the manuscript. The manuscript have also been proof read by a professional proof reader.
